# R3 PROGRAM MANAGEMENT AND WELLNESS TEAM VIEWS ON BARRIERS AND FACILITATORS TO IMPLEMENTING THE R3 PROGRAM

**DOI:** 10.1093/geroni/igz038.2853

**Published:** 2019-11-08

**Authors:** Natalie Shellito, Pamela Nadash, Edward Miller, Elizabeth Simpson, Marc Cohen

**Affiliations:** University of Massachusetts Boston, Boston, Massachusetts, United States

## Abstract

Implementing programs such as R3, which adds health-focused supportive services into senior housing sites, can be complicated. This study aimed to understand program management and wellness team views on barriers and facilitators to implementation. Semi-structured interviews were conducted with managers, social workers, and wellness nurses who implemented the R3 program. Facilitating factors included monthly phone calls between wellness team members and program participants, which strengthened relationships and provided valuable information; top-level management support, which was critical in building community partnerships; and daily ambulance reports from local emergency responders, which provided actionable information about participants. Barriers included the need for more wellness team time at individual intervention sites, challenges connecting R3 staff with participants’ hospitals and insurance companies, and refining the technological approach used to facilitate work flow and information exchange. Although obstacles were encountered during implementation, the findings provide support for the beneficial effects of enhanced services within senior housing.

